# Pharmacokinetic Properties of Rivaroxaban in Healthy Human Subjects

**DOI:** 10.7759/cureus.5484

**Published:** 2019-08-25

**Authors:** Sosipatros Bratsos

**Affiliations:** 1 Cardiology, Imperial College London, London, GBR

**Keywords:** rivaroxaban, pharmacokinetics

## Abstract

Direct oral anticoagulants (DOACs) have predictable pharmacokinetics and pharmacodynamics, limited potential for drug to drug interactions, and can be given at fixed doses without the need for routine coagulation monitoring, which makes them a very attractive alternative to vitamin K antagonists. DOACs act by specifically targeting a single coagulation factor, such as Factor Xa or thrombin. Rivaroxaban is a direct Factor Xa inhibitor and has been approved for use in several thromboembolic disorders, such as the prevention of stroke and systemic embolism in adults with non-valvular atrial fibrillation and the prevention of recurrent deep vein thrombosis and pulmonary embolism in adult patients. This review aimed to provide an overview of the mechanism of action of rivaroxaban and outline its pharmacokinetic properties (absorption, distribution, metabolism, and excretion) in healthy adult subjects.

## Introduction and background

Direct oral anticoagulants (DOACs) have recently been developed to overcome the limitations of established oral anticoagulants such as vitamin K antagonists (VKAs). In contrast to VKAs, DOACs tend to have predictable pharmacokinetics and pharmacodynamics, limited potential for drug to drug interactions and can be given at fixed doses, without the need for routine coagulation monitoring [1]. They have been shown to have similar or improved efficacy and safety with VKAs across several thromboembolic disorders and act by specifically targeting a single coagulation factor, such as Factor Xa or thrombin [2]. Factor Xa is at the convergence point of the intrinsic and extrinsic coagulation pathways and it directly converts prothrombin to thrombin via the prothrombinase complex, leading to fibrin clot formation and platelet activation [3]. Factor Xa is, thus, a promising target for effective anticoagulation. Rivaroxaban is a direct Factor Xa inhibitor and has been approved for the prevention of venous thromboembolism in adults undergoing hip or knee replacement surgery, the prevention of stroke and systemic embolism in adults with non-valvular atrial fibrillation and for the treatment of deep vein thrombosis (DVT) and pulmonary embolism (PE), as well as the prevention of recurrent DVT and PE in adult patients [2]. The aim of this review is to provide an overview of the mechanism of action of rivaroxaban and outline its pharmacokinetic properties (absorption, distribution, metabolism, and excretion) in healthy subjects.

## Review

Mechanism of action

In vitro kinetic analysis indicated that rivaroxaban is a selective, reversible, direct Factor Xa inhibitor that does not require cofactors for its anticoagulant effect [3-4]. It was found to be highly selective for human Factor Xa, with an inhibitory effect of 10,000 times higher than that for other serine proteases and an inhibition constant (K_i_) of 0.4 nM [3]. Unlike indirect Factor Xa inhibitors, such as fondaparinux, rivaroxaban inhibits clot-bound Factor Xa (half-maximal inhibitory concentration (IC_50_) 75 nM), as well as prothrombinase activity (IC_50_ 2.1 nM), thereby prolonging clotting times. In human plasma, rivaroxaban has been shown to inhibit thrombin production and, thus, the amplification processes of coagulation, through the inhibition of endogenous Factor Xa (IC_50_ of 21 nM) [3, 5]. Therapeutically relevant concentrations of rivaroxaban (80-100 nM) are sufficient to cause almost complete inhibition of thrombin generation [6]. In addition, rivaroxaban increases clot permeability and degradability, which may also contribute to its antithrombotic effect [7].

Pharmacokinetics

Absorption

A study by Kubitza et al. showed that after single-dose administration of rivaroxaban oral tablets (1.25-80 mg) in the fasted state, absorption was rapid and peak plasma concentration (C_max_) was reached after 2 hours [8]. Increases in C_max_ and area under the curve (AUC) were dose-proportional up until 10 mg doses, but less than dose-proportional above 10 mg. This might be due limited solubility causing incomplete absorption of higher doses of rivaroxaban tablets, which would be consistent with the lower proportion of drug excreted unchanged in the urine at high doses (60, 80 mg) compared to the lowest dose (1.25 mg) in this study (10% compared to 40%, respectively). However, a potential benefit of the decreased bioavailability at high doses might be the reduced risk of unintentional overdosing. Although this study was randomized, blinded, and placebo controlled, it only included 108 healthy white male subjects, aged 19 to 45 years with normal body mass index (BMI) and therefore may have been subject to selection bias, as about half of the patients on rivaroxaban are females, their median age is 74 years and their mean BMI is 28 [9]. Despite that, age, gender, ethnicity, and BMI were not shown to influence the absorption of rivaroxaban [10-12].

Another placebo-controlled and blinded dose escalation study looking at the effect of multiple-dose regimens (up to 30 mg twice daily) in 61 healthy Caucasian male subjects aged 20 to 45 years, found that C_max_ was reached 3 to 4 hours after the initial administration for all doses and all regimens and that at steady state [13]. Nevertheless, the AUC and C_max_ were dose-proportional for all doses, as opposed to the above study [8]. However, in this study rivaroxaban was taken in the fed state. The small sample size and possible selection bias limited the accuracy of the results. Another weakness could be the unequal randomization of patients to either drug or placebo (2:1 ratio), as it is possible that when outcome assessors are not agnostic about allocation (there is higher probability that a patient will fall under the intervention group), both positive and negative events might be altered [14].

The effect of food on the absorption and pharmacokinetics of rivaroxaban was investigated in a set of six independent, single-dose, cross-over studies in healthy male subjects [15]. Absorption was shown to be almost complete, with high oral bioavailability of greater than 80% in doses up to 10 mg, while the AUC and C_max_ were dose-proportional regardless of fasted or fed condition­s. However, under fasting conditions, the pharmacokinetic parameters of 15 mg and 20 mg increased with dose but were less than dose-proportional, with decreased bioavailability and absorption rate with increasing dose, consistent with Kubitza et al. [8]. For example, bioavailability dropped to 66% for the 20 mg dose under fasting conditions. When rivaroxaban was taken with food however, dose proportionality and high bioavailability (more than 80%) of these high doses were achieved. For instance, the mean AUC and C_max_ of the 20 mg dose increased by 39% and 76%, respectively with food compared to fasted. The increased absorption in the fed state might relate to the lipophilicity and limited aqueous solubility of rivaroxaban. Finally, the absorption of rivaroxaban was unaffected by drugs altering gastric pH, such as H2-receptor antagonists or antacids [16].

Distribution

In vitro protein binding in human plasma for rivaroxaban was found to be high (95%) and reversible [17]. Experiments on isolated human plasma proteins revealed that serum albumin was the most significant plasma binding component (free fraction of rivaroxaban, f_u_, was 19.6%) and then a_1_-acid glycoprotein (f_u_ = 67.7%). The discrepancy between the fuin human plasma (5.1%) and in human serum albumin (19.6%) could be explained by the presence of free fatty acids, whose binding might cause conformational changes in the albumin structure, therefore, increasing the protein binding of rivaroxaban. Moreover, a concentration-dependent increase in the fuof rivaroxaban was observed at concentrations above 10 mg/L, possibly due to saturation of binding sites. However, the above in vitro results may not accurately predict what happens in vivo. The apparent volume of distribution (V_ss_) was estimated at 55 L from a population modeling analysis of rivaroxaban’s pharmacokinetics, which used data from young, healthy subjects included in phase I trials, also indicating that it is mainly distributed to plasma, with a moderate affinity to peripheral tissues [18]. The accuracy of the model and its ability to provide mean population estimates is, however, debatable, given the small amount of data used. Rivaroxaban’s moderate tissue affinity could be due to its moderate lipophilicity, as reflected in its partition coefficient value (logP=1.5) [1]. This might explain the insignificant effect of body weight on the pharmacokinetics of rivaroxaban (and thus the fixed-dosing regimens regardless of body weight), as it is unlikely that total vascular bed size and blood volume will vary as much between subjects as body weight itself, which is mainly affected by the amount of body fat [11].

Metabolism

The metabolism of rivaroxaban was evaluated by Weinz et al. with the use of high-performance liquid chromatography and radioactivity detection by liquid scintillation counting [19]. The percentage of the initial oral dose that was found to undergo metabolic degradation was 57%. Rivaroxaban was metabolized by both cytochrome P450 enzymes (CYP3A4/5, CYP2J2) and CYP-independent mechanisms. CYP3A4 accounted for 18% and CYP2J2 for 14% of total rivaroxaban elimination [19-20]. In addition to this oxidative biotransformation, non-CYP-mediated hydrolysis of the amide bonds accounted for 14% of total rivaroxaban elimination. No major or active metabolites were detected in plasma and unchanged rivaroxaban was the most important compound in human plasma (89% of total AUC), reflecting a minimal presence of metabolites. In fact, the plasma concentration-time curve for unchanged rivaroxaban was very similar to that for total radioactivity, meaning that the pharmacological effects of rivaroxaban were largely due to the unchanged drug. The predominant metabolite M-1 accounted for only 3% of total plasma radioactivity. In this study, however, only four young male subjects of normal BMI were used, limiting the credibility and perhaps the generalisability of the findings. In vitro structural studies in liver microsomes and hepatocytes of humans showed that M-1 was produced through oxidative degradation of the morpholinone moiety of rivaroxaban (forming M2), followed by oxidative cleavage of the ring [20]. Oxidative degradation of the morpholinone ring also produced the minor metabolites M-5 and M-6. Metabolite M-4 was produced by hydrolysis of the amide bond of rivaroxaban, with subsequent conjugation of M-13 with glycine. Hydrolytic cleavage of the lactam amide bond in the morpholinone moiety resulted in M-7. NADPH-independent hydrolysis of the amide bond of rivaroxaban formed M-15, which underwent NADPH-independent oxidation to form M-16 via monoamine oxidases in human liver microsomes. M-16 was then either reduced to the alcohol derivative M-17 or further oxidized to the carboxylic acid derivative M-18. Clearly, in vitro studies are inherently flawed by nature, as they may not accurately describe the metabolism in vivo. For example, this study excluded the possibility that extra-hepatic enzymes might play a role in the metabolic degradation of rivaroxaban, since only hepatocytes or liver microsomes were used.

In summary, the metabolism of rivaroxaban was shown to occur via two major pathways: oxidative degradation of the morpholinone moiety (via CYP450) and hydrolysis of the different amide bonds (CYP-independent). Given then role of CYP450 in rivaroxaban’s metabolism, it would be reasonable to assume that inhibitors or inducers of these enzymes would affect the drug’s pharmacokinetics. Indeed, Mueck et al. demonstrated that concomitant administration of rivaroxaban with strong inhibitors of both CYP3A4 and P-glycoprotein/breast cancer resistance protein (P-gp/BCRP) (such as ketoconazole and ritonavir), significantly increased rivaroxaban exposure [21]. By inhibiting CYP3A4, the metabolism of rivaroxaban will be reduced and thus the plasma concentration of the active, unchanged drug will be increased. However, strong inhibitors of either CYP3A4 or P-gp, or moderate inhibitors of both of these pathways, produced less marked effects. Finally, due to the highly polymorphic nature of CYP450s, a degree of inter-individual variability is expected in the metabolism of rivaroxaban, but more studies are necessary to evaluate the magnitude of the effect of CYP450 single nucleotide polymorphisms on rivaroxaban’s pharmacokinetics [22].

Excretion

Weinz et al. showed that excretion occurred predominantly via the renal route (66%) and to a lesser extent via the faecal/biliary routes (28%), by measuring rivaroxaban associated radioactivity in humans [19]. The percentage of the initial dose excreted as unchanged drug was 43%, of which 36% was excreted via urine and 7% via the feces. Of the 36% of the rivaroxaban dose eliminated in urine, active renal secretion accounted for approximately 30% and glomerular filtration for the rest [18-19]. According to in vitro and in vivo drug studies, the transporters involved in active renal secretion of rivaroxaban are P-gp and BCRP. Inhibiting P-gp/BCRP would therefore result in reduced renal clearance and enhanced drug exposure, as discussed above [21, 23]. In addition, 30% and 21% of the initial dose was eliminated as metabolites in the urine and feces, respectively. Mean terminal half-life of about six to nine hours and apparent oral clearance of about 10 L/hour were found in young healthy subjects in doses up to 20 mg [8, 13]. In a study of healthy subjects of 75 years old and over however, half-life was found to be 11 to 12 hours, whereas total and renal clearance inversely correlated with age, but positively with creatinine clearance, which is age-dependent and a marker of renal function [10]. The reduced rivaroxaban clearance in the elderly subjects, as a result of the expected and well-documented reduction in renal function with increasing age, led to a statistically significant impact on the exposure of rivaroxaban [24]. Nevertheless, dose-adjustments based on age alone would not be necessary, as age was insufficient to explain the increased drug exposure on its own, but may be required based on renal function. A limitation of this study was once again the small sample size (n=34), but an important strength in contrast to the other studies, was the equal representation of both genders, as well as the greater range of BMIs. The above is in agreement with studies in patients with renal impairment, which have demonstrated that renal impairment results in decreased renal clearance of a single rivaroxaban 10 mg oral dose and increased overall exposure to the drug [25].

## Conclusions

In this review, the mechanism of action and the pharmacokinetic properties of rivaroxaban in healthy subjects were outlined. Rivaroxaban is a direct, reversible and highly selective factor Xa inhibitor that inhibits thrombin generation at therapeutically relevant concentrations. Overall, in healthy subjects, rivaroxaban was shown to exhibit predictable and dose-proportional pharmacokinetics in the fed state without dose accumulation beyond a steady state. Demographic variations had little influence on rivaroxaban’s pharmacokinetics, except for age, but its effect was found to be insignificant after adjusting for renal function. Furthermore, co-administration of rivaroxaban with strong inhibitors of both CYP3A4 and P-gp was identified to cause a clinically relevant increase in drug exposure, which was expected given their role in the drug’s metabolism and excretion. Finally, it is important to note that, since this review aimed to examine the pharmacokinetics of rivaroxaban in healthy volunteers, the majority of the studies evaluated were phase I and had significant limitations, as discussed. To summarise, the high oral bioavailability, the low-to-moderate potential for peripheral tissue penetration, combined with the absence of major or active metabolites in human plasma and its relatively short half-life, highlight rivaroxaban’s favorable pharmacokinetics and justify its central role in current anticoagulation management across several thromboembolic conditions.
